# Response is increased using postal rather than electronic questionnaires – new results from an updated Cochrane Systematic Review

**DOI:** 10.1186/s12874-024-02332-0

**Published:** 2024-09-16

**Authors:** Phil Edwards, Chloe Perkins

**Affiliations:** Data Collection Methodology Group, Department of Population Health, Faculty of Epidemiology and Population Health, London School of Hygiene & Tropical Medicine, Keppel Street, London, WC1E 7HT UK

**Keywords:** Data collection, Questionnaires and surveys, Survey methodology, Nonrespondents, Self report

## Abstract

**Background:**

A decade ago paper questionnaires were more common in epidemiology than those administered online, but increasing Internet access may have changed this. Researchers planning to use a self-administered questionnaire should know whether response rates to questionnaires administered electronically differ to those of questionnaires administered by post. We analysed trials included in a recently updated Cochrane Review to answer this question.

**Methods:**

We exported data of randomised controlled trials included in three comparisons in the Cochrane Review that had evaluated hypotheses relevant to our research objective and imported them into Stata for a series of meta-analyses not conducted in the Cochrane review. We pooled odds ratios for response using random effects meta-analyses. We explored causes of heterogeneity among study results using subgroups. We assessed evidence for reporting bias using Harbord’s modified test for small-study effects.

**Results:**

Twenty-seven trials (66,118 participants) evaluated the effect on response of an electronic questionnaire compared with postal. Results were heterogeneous (I-squared = 98%). There was evidence for biased (greater) effect estimates in studies at high risk of bias; A synthesis of studies at low risk of bias indicates that response was increased (OR = 1.43; 95% CI 1.08–1.89) using postal questionnaires. Ten trials (39,523 participants) evaluated the effect of providing a choice of mode (postal or electronic) compared to an electronic questionnaire only. Response was increased with a choice of mode (OR = 1.63; 95% CI 1.18–2.26). Eight trials (20,909 participants) evaluated the effect of a choice of mode (electronic or postal) compared to a postal questionnaire only. There was no evidence for an effect on response of a choice of mode compared with postal only (OR = 0.94; 95% CI 0.86–1.02).

**Conclusions:**

Postal questionnaires should be used in preference to, or offered in addition to, electronic modes.

**Supplementary Information:**

The online version contains supplementary material available at 10.1186/s12874-024-02332-0.

## Introduction

### Rationale

When collecting information from large, geographically dispersed populations, a self-administered questionnaire is usually the only financially viable option [1]. Non-responses to questionnaires reduce effective sample sizes, reducing study power, and may introduce bias in study results [2]. The Cochrane Methodology Review of methods to increase response to self-administered questionnaires has provided a much-used scientific evidence base for effective data collection by questionnaire since the publication of the first version of the review in 2003 which focused on postal questionnaires [3]. 

A decade ago paper-and-pencil administration of questionnaires in epidemiological studies was twenty times more common than electronic administration [4], but increased Internet access and decreasing volumes of mailed letters suggests that electronic administration has gained favour [5–7]. Researchers planning to collect data from participants using a self-administered questionnaire need to know how will the proportion of participants responding to a questionnaire administered electronically compare with one administered by post? We conducted further analyses of the trials included in the recently updated Cochrane Review [8] to answer this question.

### Objective

To assess whether response rates to questionnaires administered electronically differ to those of questionnaires administered by post.

## Methods

### Data sources/measurement

We exported data of randomised controlled trials included in the updated Cochrane Review [8] from RevMan and imported them into Stata for a series of meta-analyses not conducted in the Cochrane review.

#### Comparisons

We focused on data from trials included in three comparisons in the Cochrane Review that had evaluated hypotheses relevant to our research objective:

1. Postal vs. electronic questionnaire (Cochrane Comparison 81).
2. Electronic questionnaire only vs. choice (postal or electronic) (Cochrane Comparison 84).
3. Choice (electronic or postal) vs. postal questionnaire only (Cochrane Comparison 82).

These comparisons assess: response to questionnaires administered by post compared with questionnaires administered electronically, response to a questionnaire administered electronically compared with response when including a postal response option, and response when including an electronic response option compared with response to a questionnaire administered by post only, respectively.

### Data items

#### Outcome measures

The data obtained from each trial included the numbers of participants randomised to each arm of the trial with the numbers of completed, or partially completed questionnaires returned after all mailings (for trials including a postal questionnaire), and the numbers of participants randomised to each arm of the trial with the numbers of participants submitting the completed, or partially completed online questionnaires after all contacts (electronic questionnaire).

#### Other variables

Additional data were also extracted on the:

- Year of publication of the study.
- Risk of bias in each included study (a judgment - high, low, or unclear); we assessed the overall risk of bias in each study using the Cochrane Collaboration’s tool [9].

### Effect measures and synthesis

For each of the three comparisons (2.1.1 above), we pooled the odds ratios for response in each included study in a random effects meta-analysis (to allow for heterogeneity of effect estimates between studies) using the metan command in Stata [10]. This command also produced a forest plot (a visual display of the results of the individual studies and syntheses) for each comparison. We quantified any heterogeneity using the *I*^2^ statistic that describes the percentage of the variability in effect estimates that is due to heterogeneity [11]. 

### Subgroup analyses

We explored possible causes of heterogeneity among study results by conducting subgroup analyses according to two study-level factors: Year of study publication, and risk of bias in studies. We used a statistical test of homogeneity of the pooled effects in subgroups to assess evidence for subgroup differences. The statistical test of homogeneity used is Cochran’s Q test, where the Q statistic is distributed as a chi-square statistic with k-1 degrees of freedom, where k is the number of subgroups. If there was evidence for subgroup differences provided by the test of homogeneity, we chose the ‘best estimate of effect’ as the estimate from the subgroup of studies with low risk of bias, or the subgroup of studies published after 2012. If there was no evidence for subgroup differences, we chose our best estimate of effect based on the synthesis of all studies.

#### Year of study publication

From 2012, household access to a computer exceeded 40%: [5] As the odds ratios for response to questionnaires administered electronically may be associated with household access to a computer, we analysed trial results in two subgroups – before 2012 and after 2012, where we used the year of publication as an approximation of the year of study conduct.

#### Risk of bias

The odds ratios for response estimated in the included studies may be associated with trial quality. [12, 13] For this reason we analysed trial results in two subgroups – trials judged to be at low and at high risk of bias.

### Reporting bias assessment

We assessed evidence for reporting bias using Harbord’s modified test for small-study effects implemented in Stata using the metabias command [14]. This test maintains better control of the false-positive rate than the test proposed by Egger at al [14]. 

## Results

### Study characteristics

Thirty-five studies [15–49] reported 45 independent trials included in one or more of the three comparisons (Table 1). The studies were conducted in the US (*n* = 20), Europe (*n* = 13), and Australasia (*n* = 2). The studies included between 133 and 12,734 participants and were published between 2001 and 2020. Eight studies were judged to be at high risk of bias [16, 19, 33, 34, 42, 43, 45, 46].

**Table 1:** Characteristics of 35 included studies

First author	Year	Setting	Comparison	Study size	Participants	Risk of Bias							Topic	Questionnaire description
						SG^±^	AC^∀^	BPP^¥^	BOO^§^	IOD^&^	SR^€^	OSB^α^		
Akl	2005	US	1. Postal vs. Electronic questionnaire	202	Residents and faculty of the University at Buffalo Internal Medicine Residency program	Unclear	Unclear	Unclear	Low	Low	Low	Low	Academic issues	28 questions (residents), 23 (faculty).
Basnov	2009	Denmark	1. Postal vs. Electronic questionnaire	782	Women referred for mammography	Unclear	Unclear	Unclear	Low	High	High	Low	Anxiety and Depression	Short Form Health Survey and Hospital Anxiety and Depression Scale − 17 pages, 119 items.
Bech	2009	Denmark	1. Postal vs. Electronic questionnaire	9,900	People aged 50–75 years drawn from the Central National Population Register	Unclear	Unclear	Unclear	Low	Low	Low	Low	Design of nursing homes and facilities	35 questions; 16 pages
Beebe	2018	US	1. Postal vs. Electronic questionnaire	342	Primary care clinicians, including physicians, nurse practitioners, and physician assistants	Unclear	Unclear	Unclear	Low	Low	Low	Low	Clinician knowledge, barriers, and perceived parental barriers regarding HPV vaccination.	Questionnaire not described
Bergeson	2013	US	1. Postal vs. Electronic questionnaire	9,313	Patients receiving care at 6 clinics in Minnesota.	Unclear	Unclear	Unclear	Low	High	Low	Low	Patient experiences with care.	CAHPS hybrid survey, 22 questions
Bjertnaes	2018	Norway	1. Postal vs. Electronic questionnaire; 2. Electronic only vs. Choice (postal or electronic); 3. Choice (electronic or postal) vs. postal only	1,696	Parents of children with type 1 diabetes in the Norwegian Childhood Diabetes Registry with at least one outpatient consultation in the previous year.	Unclear	Unclear	Unclear	Low	Low	Low	Low	Parent experiences with their child’s diabetes outpatient care.	40 questions, 4 pages.
Bray	2017	UK	2. Electronic only vs. Choice (postal or electronic)	8,765	Young people in the Avon Longitudinal Study of Parents and Children (ALSPAC) birth cohort study	Low	Low	Low	Low	Low	Low	Low	Gambling, Self-Harm, Employment, Education & Training, Tobacco & Alcohol.	A5 booklet of 44 pages; Online questionnaire designed to be similar to the paper questionnaire.
Brøgger	2007	Norway	3. Choice (electronic or postal) vs. postal only	4,213	People aged 20–40 years from the Norwegian Central Population Registry.	Unclear	Unclear	Low	Low	Low	Low	Low	Asthma and allergies	one-page, 40 questions
Clark	2011	US	1. Postal vs. Electronic questionnaire	205	Director of Nursing and Administrator in nursing homes with at least 30 beds.	Unclear	Unclear	Unclear	Low	Low	Low	Low	Q55uestionnaire not described	Questionnaire not described
Cobanoglu	2001	US	1. Postal vs. Electronic questionnaire	194	Hospitality professors with email addresses from members of the Council on Hotel, Restaurant, and Institutional Education.	Unclear	Unclear	Unclear	Low	Low	Low	Low	Hospitality education.	Questionnaire not described
Fluss	2014	Scotland, UK	1. Postal vs. Electronic questionnaire	4,417	Residents in Grampian (north of Scotland, UK) aged 25+.	Low	Low	Unclear	Low	Low	Low	Low	Pain and pain management	20-pages.
Fowler	2019	US	1. Postal vs. Electronic questionnaire	4,323	Patients age 18 + with at least one primary care visit during previous 6 months, Boston.	Unclear	Unclear	Unclear	Low	High	Low	Low	Medical care experiences.	56 questions, standard CAHPS survey, 12 pages.
Hardigan	2012	US	1. Postal vs. Electronic questionnaire; 2. Electronic only vs. Choice (postal or electronic); 3. Choice (electronic or postal) vs. postal only	3,953	Dentists from the Florida Department of Health, Board of Dentistry.	Unclear	Unclear	Unclear	Low	Low	Low	Low	Tobacco use by dental patients	28 questions; 4 pages.
Hardigan	2016	US	1. Postal vs. Electronic questionnaire	4,661	Practicing pharmacists.	Unclear	Unclear	Unclear	Low	Low	Low	Low	Job satisfaction.	60 questions; 4 pages.
Hohwu	2013	Denmark	2. Electronic only vs. Choice (postal or electronic); 3. Choice (electronic or postal) vs. postal only	1,573	Parents of children aged 2–17 years	Unclear	Unclear	Unclear	Low	Low	Low	Low	Children’s health and welfare	NordChild questionnaire, 28 pages, 73 questions.
Iversen	2020	Norway	2. Electronic only vs. Choice (postal or electronic)	6,454	Patients aged 16 + registered with a GP.	Unclear	Unclear	Unclear	Low	Low	Low	Low	User experiences with healthcare.	47 questions on 6 pages.
Jacob	2012	US	1. Postal vs. Electronic questionnaire	556	High school principals, Michigan.	Unclear	Unclear	Unclear	Low	Low	Low	Low	Online learning and virtual education	42 questions; 15 min completion time
Lagerros	2012	Sweden	1. Postal vs. Electronic questionnaire	393	People from the population register.	Unclear	Unclear	Unclear	Low	Low	Low	Low	Physical activity.	4 questions.
Leece	2004	US	1. Postal vs. Electronic questionnaire	442	Surgeon-members of the Orthopaedic Trauma Association	High	High	Unclear	Low	Low	Low	Low	Operative treatment for femoral neck fractures.	8 pages
Mauz	2018	Germany	1. Postal vs. Electronic questionnaire; 3. Choice (electronic or postal) vs. postal only;	6,194	Children and adolescents participating in the third wave of the ‘German Health Interview and Examination Survey for Children and Adolescents’.	Unclear	Unclear	Unclear	Low	High	Low	Low	Health care utilisation, living conditions, and environmental determinants of health.	Questionnaire not described
Millar	2011	US	1. Postal vs. Electronic questionnaire; 2. Electronic only vs. Choice (postal or electronic); 3. Choice (electronic or postal) vs. postal only	1,345	Undergraduate students at the main campus of Washington State University.	Unclear	Unclear	Unclear	Low	Low	Low	Low	Experiences at University.	36 questions.
Millar	2019	US	1. Postal vs. Electronic questionnaire	470	Adults with colorectal, breast, prostate, ovarian, and multiple myeloma cancers, reported in the Utah Cancer Registry.	Unclear	Unclear	Unclear	Low	Low	Low	Low	Cancer recurrence and willingness to participate in cancer research.	35 questions.
Murphy	2020	US	2. Electronic only vs. Choice (postal or electronic);	12,734	Physicians from the American Medical Association MasterFile, from haematology or oncology specialties.	Low	Low	Low	Low	Low	Low	Low	Referral and recruitment of patients to clinical trials and barriers to trial accrual.	5-min survey
Reinisch	2016	US	1. Postal vs. Electronic questionnaire	309	Members of the American Association of Plastic Surgeons	Unclear	Unclear	Unclear	Low	Low	Low	Low	Authorship issues.	Questionnaire not described
Sakshaug	2019	Germany	1. Postal vs. Electronic questionnaire	8,996	Public & private establishments that had previously participated in a Job Vacancy Survey and were registered as employing staff in at least one of 25 target professions.	Unclear	Unclear	Unclear	Low	Low	Low	Low	Factors that influence establishments’ decision-making process for filling job vacancies.	Questionnaire not described
Schmuhl	2010	US	1. Postal vs. Electronic questionnaire; 2. Electronic only vs. Choice (postal or electronic); 3. Choice (electronic or postal) vs. postal only	1,178	Emergency medical services (EMS) providers with current certification in Utah.	Unclear	Unclear	Low	Low	Low	Low	Low	Utah Emergency Services for Children needs assessment.	31 questions.
Schwartzenberger	2017	US	1. Postal vs. Electronic questionnaire	646	Patients who underwent endoscopic carpal tunnel release	Unclear	Unclear	Unclear	Low	Low	Low	Low	Patient-reported outcomes 1 year following carpal tunnel release	23 questions
Scott	2011	Australia	2. Electronic only vs. Choice (postal or electronic)	1,741	Doctors undertaking clinical practice drawn from a national directory of doctors.	Low	Low	High	Low	Low	Low	Low	Balancing Employment & Life -Workforce participation and its determinants among Australian doctors.	58 questions, 8 page booklet (specialists in training); 87 questions, 13 page booklet (specialists).
Sebo	2017	Switzerland and France	1. Postal vs. Electronic questionnaire	3,400	Community-based GPs with a valid and available email address	Low	Low	Unclear	Low	High	Low	Low	GPs’ preventive care activities.	37 questions.
Taylor	2019	US	1. Postal vs. Electronic questionnaire	133	Individuals aged 6–24 years with smartphones, recruited via Internet to participate in a study about mood and mobile game habits.	Unclear	Unclear	Low	Low	Low	Low	Low	Mood, mobile games, and the weather.	6 daily multiple-choice questions
van den Berg	2011	Holland	2. Electronic only vs. Choice (postal or electronic)	277	Female survivors of childhood cancer	High	High	Unclear	Low	Low	Low	Low	Reproductive function, ovarian reserve, and risk of premature menopause in female childhood cancer survivors.	Department of Epidemiology of the Netherlands Cancer Institute Questionnaire in a cohort study on long-term effects of ovarian stimulation for in vitro fertilization.
Weaver	2019	US	1. Postal vs. Electronic questionnaire	636	Physicians from the Minnesota Board of Medical Practice with both a postal and email address listed.	Unclear	Unclear	Unclear	Low	High	Low	Low	Factors that influence physicians’ willingness to disclose medical errors and adverse events to patients and their families.	Questionnaire not described
Whitehead	2011	New Zealand	1. Postal vs. Electronic questionnaire	1,914	Students randomly selected from a database containing all university students.	Low	Low	Unclear	Low	Low	Low	Low	Anxiety and depression in non-psychiatric populations.	41 items.
Yetter	2010	US	1. Postal vs. Electronic questionnaire	780	School psychologists with an email address.	Unclear	Unclear	Unclear	Low	Low	Low	Low	Perceptions of Pre-referral Intervention Teams for addressing children’s school-related academic and behavior difficulties.	66 items.
Ziegenfuss	2010	US	3. Choice (electronic or postal) vs. postal only	770	Randomly selected Olmsted County residents aged 25–65.	Unclear	Unclear	Unclear	Low	Low	Low	Low	Bowel disease.	16 pages

Risk of Bias key: ± Sequence generation ∀ Allocation concealment ¥ Blinding of participants and personnel § Blinding of outcome assessment & Incomplete outcome data € Selective reporting α Other sources of bias

### Results of syntheses

#### Comparison 1 - Postal vs. electronic questionnaire

**Fig. 1:**
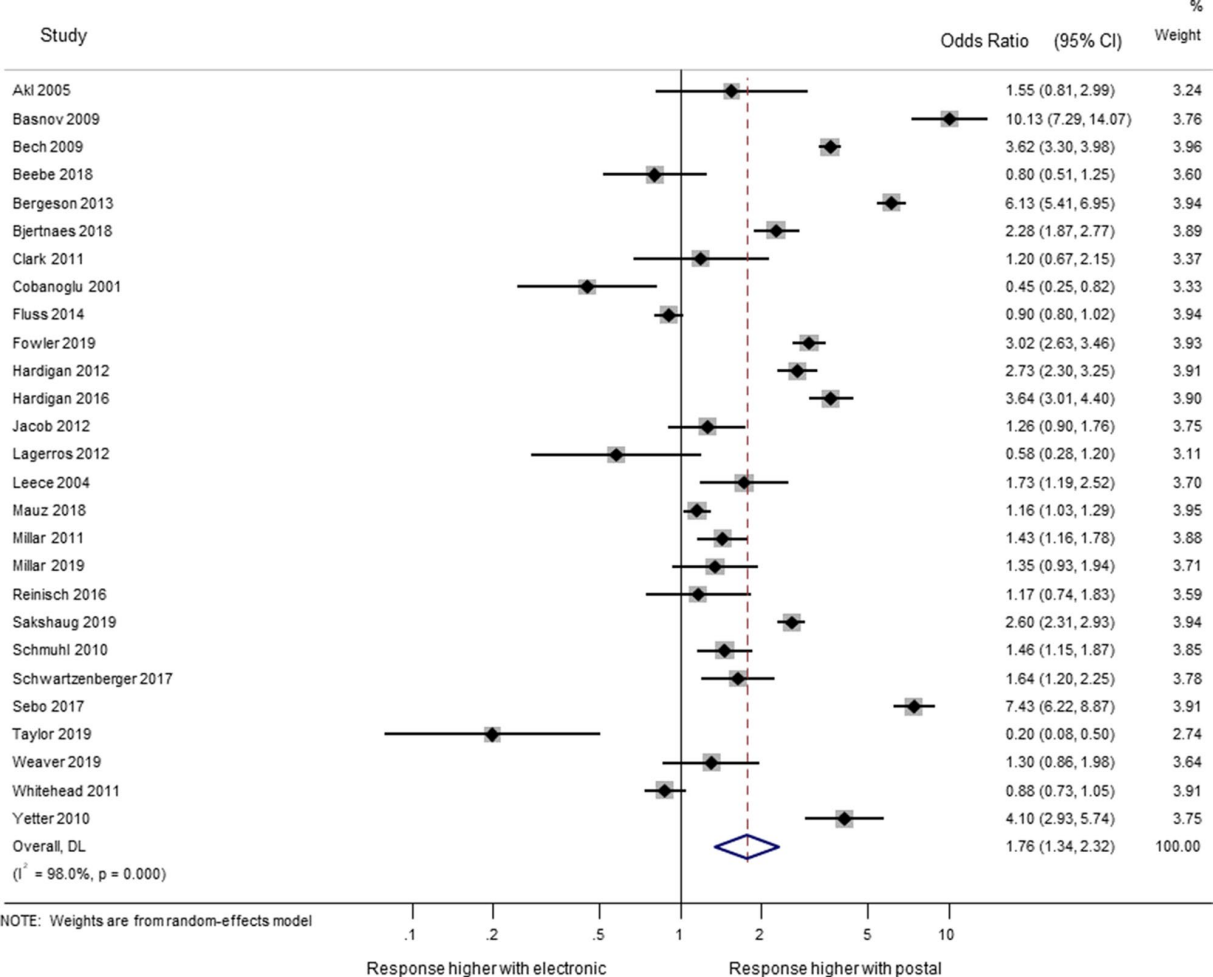
Effect on response of mode of administration

Twenty-seven trials (66,118 participants) evaluated the effect on questionnaire response of postal administration compared with electronic. [15–20, 23–28, 31–36, 38–41, 43, 44, 46–48] The odds of response were increased by over half (OR 1.76; 95% CI 1.34 to 2.32) using a postal questionnaire when compared with an electronic one (Fig. 1). There was considerable heterogeneity between the trial results (I-squared = 98%), but most of the studies showed response was greater with postal questionnaires than with electronic questionnaires, and the high I-squared is due to differences in the size of the benefit for postal questionnaires, rather than being due to an even spread of results between those favouring postal and those favouring electronic questionnaires.

#### Comparison 2 - electronic questionnaire only vs. choice (postal or electronic)

**Fig. 2:**
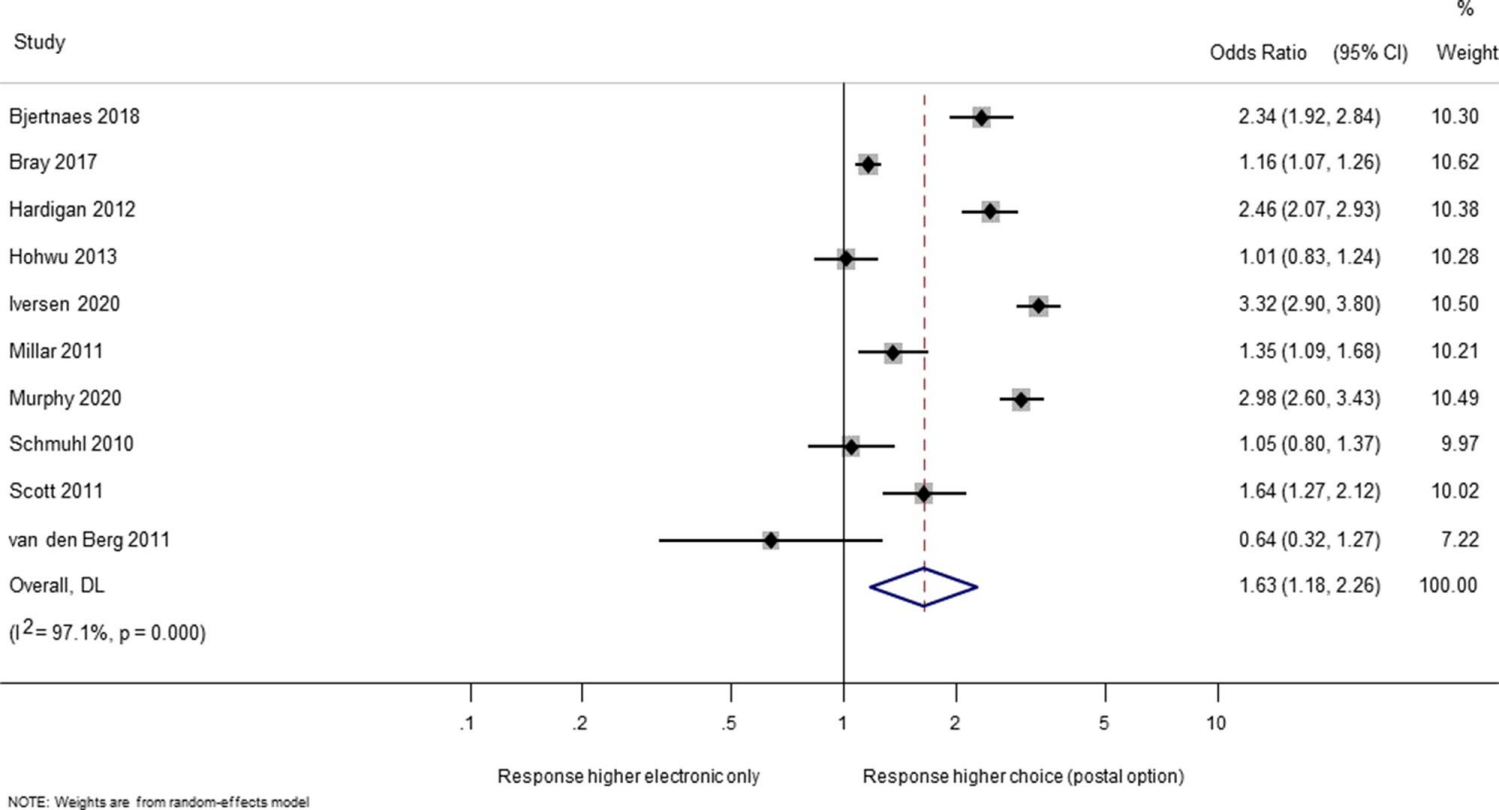
Effect on response of choice of response mode compared with electronic only

Ten trials (39,523 participants) evaluated the effect on questionnaire response of providing a choice of response mode (postal or electronic) compared to an electronic questionnaire only [20, 21, 27, 29, 30, 35, 37, 40, 42, 45]. The odds of response were increased by over half when providing a choice of response mode (OR 1.63; 95% CI 1.18 to 2.26; Fig. 2). There was considerable heterogeneity between the trial results (I-squared = 97.1%), but again, most of the studies favoured giving people the choice of response mode rather than electronic questionnaire only, and the high I-squared is due to differences in the size of the benefit for choice, rather than being due to an even spread of results between those favouring choice and those favouring electronic only.

#### Comparison 3 - choice (electronic or postal) vs. postal only

**Fig. 3:**
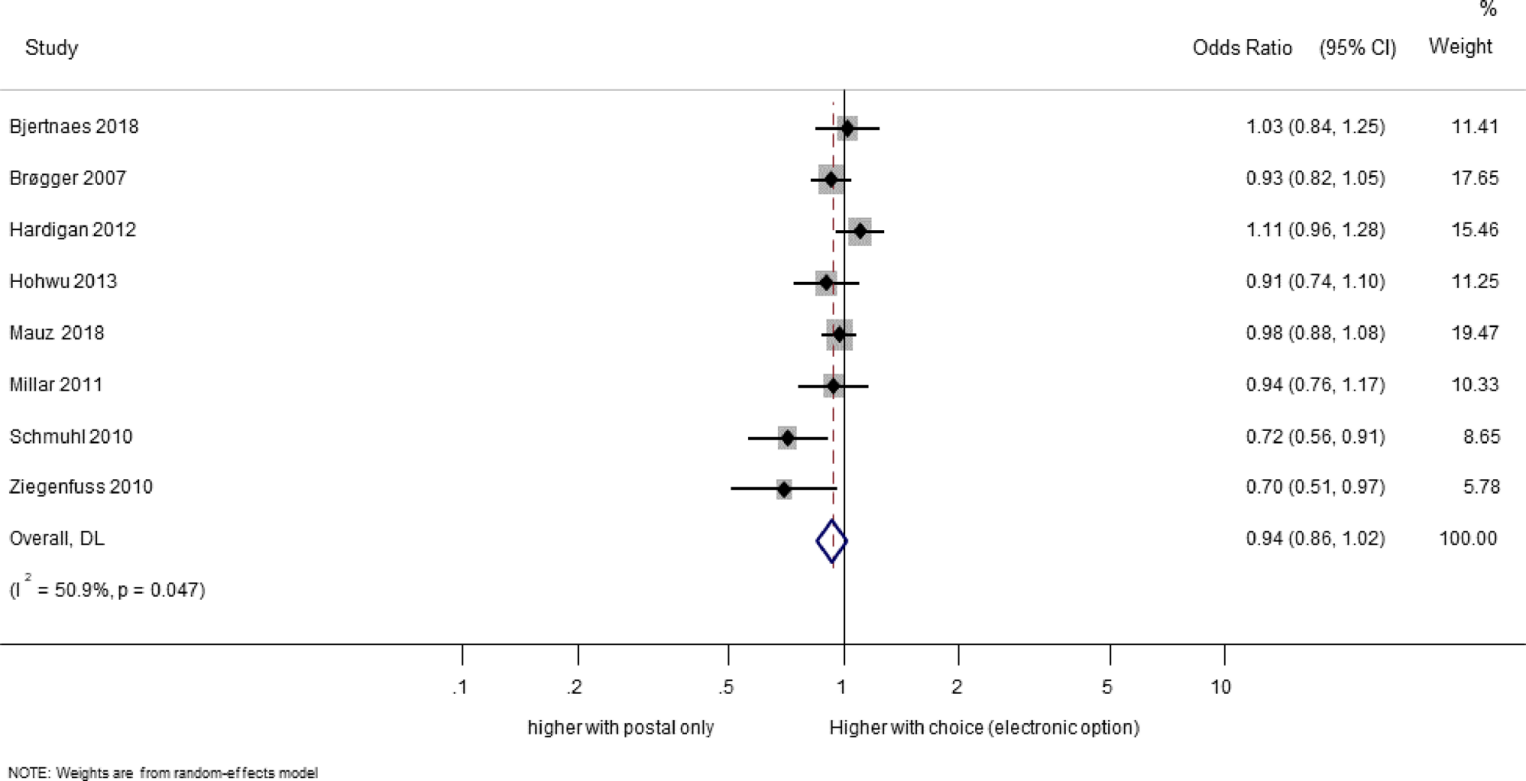
Effect on response of choice of response mode compared with postal only

Eight trials (20,909 participants) evaluated the effect of providing a choice of response mode (electronic or postal) compared to postal response only [20, 22, 27, 29, 34, 35, 40, 49]. There was no evidence for an effect on response of providing a choice (OR 0.94; 95% CI 0.86 to 1.02; Fig. 3). There was moderate heterogeneity among the trial results (I-squared = 50.9%).

### Results of subgroup analyses

Table 2 presents the results of subgroup analyses according to the two study-level factors (forest plots of these subgroup analyses are included in supplementary figures).

**Table 2:** Results of subgroup analyses of according to two study-level factors

Comparison 1		Number of studies	OR	95%CI	I-squared
Subgroups	All studies	27	1.76	1.34–2.32	98%
*Year of publication*	before 2012	10	1.85	1.12–3.06	97.5%
	after 2012	17	1.70	1.19–2.43	98.3%
*Risk of Bias*	High	7	3.24	1.68–6.25	99%
	Low	20	1.43	1.08–1.89	96.8%
Comparison 2		Number of studies	OR	95%CI	I-squared
Subgroups	All studies	10	1.63	1.18 to 2.26	97.1%
*Year of publication*	before 2012	4	1.22	0.93–1.61	69.6%
	after 2012	6	2.02	1.30–3.13	98.2%
*Risk of Bias*	High	2	1.08	0.43–2.71	84.2%
	Low	8	1.77	1.23–2.55	97.7%
Comparison 3		Number of studies	OR	95%CI	I-squared
Subgroups	All studies	8	0.94	0.86–1.02	50.9%
*Year of publication*	before 2012	4	0.85	0.73–0.98	48.5%
	after 2012	4	1.01	0.93–1.08	7.0%
*Risk of Bias*	High	1	0.98	0.88–1.08	-
	Low	7	0.92	0.83–1.03	57.1%

#### Comparison 1 - postal vs. electronic questionnaire

##### Year of publication

A third of studies were published before 2012 [15–17, 23, 24, 33, 35, 40, 47, 48]. In this subgroup of studies the odds of response were 85% greater (OR 1.85; 95% CI 1.12 to 3.06) with a postal questionnaire compared with an electronic one. In the subgroup of studies published after 2012 the effect was lower (OR 1.70; 1.19 to 2.43), consistent with our concern (Sect. 2.4.1) that higher household access to a computer from 2012 may have improved preference for electronic questionnaires, however the statistical test of homogeneity of the pooled effects in these two subgroups was not significant (*p* = 0.788), indicating no evidence from these studies for different effects by year of study (Supplementary Fig. 1a).

##### Risk of bias

Seven of the trials [16, 17, 26, 33, 34, 43, 46] were judged to be at high risk of bias and for these trials the odds of response were more than tripled (OR 3.24; 95% CI 1.68 to 6.25) using a postal questionnaire when compared with an electronic one. There was considerable heterogeneity between the trial results (I-squared = 99%).

When only the 20 trials deemed to be at low risk of bias were synthesised, the odds of response were increased by two-fifths (OR 1.43; 95% CI 1.08 to 1.89). There was also considerable heterogeneity between these trial results (I-squared = 96.8%).

The statistical test of homogeneity of the pooled effects in these two subgroups (*p* = 0.025) provides some evidence for greater effect estimates in studies at high risk of bias (Supplementary Fig. 1b). Our best estimate of the effect on response of mode of administration is hence from a synthesis of the studies at low risk of bias (OR 1.43; 95% CI 1.08 to 1.89). Results overall were thus confounded by risk of bias, but this did not explain the between study heterogeneity.

#### Comparison 2 - electronic questionnaire only vs. choice (postal or electronic)

##### Year of study

Half of studies were published before 2012 [35, 40, 42, 45]. In this subgroup of studies there was no evidence for an effect on response of providing a postal response option (OR 1.22; 95% CI 0.93 to 1.61). In the subgroup of studies published after 2012 there was evidence for an effect on response of providing a postal response option (OR 2.02; 95% CI 1.30 to 3.13). The statistical test of homogeneity of the pooled effects in these two subgroups was significant (*p* = 0.057), indicating some evidence from these studies for different effects by year of study (Supplementary Fig. 2a). This apparent preference for a postal response option in studies published after 2012 was counter to our concern (Sect. 2.4.1) that higher household access to a computer from 2012 would improve preference for electronic questionnaires. There was considerable heterogeneity between the trial results (I-squared = 98.2%), but most of the studies favoured giving people the choice of response mode rather than electronic questionnaire only, and the high I-squared is due to differences in the size of the benefit for choice, rather than being due to an even spread of results between those favouring choice and those favouring electronic only.

##### Risk of bias

Two of the trials were judged to be at high risk of bias [42, 45]. There was no evidence for an effect on response of a postal option in these studies (OR 1.08; 95% CI 0.43 to 2.71). When only the 8 trials deemed to be at low risk of bias were synthesised, there was evidence that the odds of response were increased when providing a postal response option (OR 1.77; 95% CI 1.23 to 2.55). There was considerable heterogeneity between these trial results (I-squared = 97.7%). The statistical test of homogeneity of the pooled effects in these two subgroups (*p* = 0.326) provides no evidence for different effects by risk of bias (Supplementary Fig. 2b). Our best estimate of the effect on response of providing a postal response option is hence from a synthesis of all of these studies (OR 1.63; 95% CI 1.18 to 2.26).

#### Comparison 3 - choice (electronic or postal) vs. postal questionnaire only

##### Year of study

In the subgroup of studies published before 2012 there was very weak evidence that the odds of response were lower with an electronic option (OR 0.85; 0.73 to 0.98), whereas in studies published after 2012 there was no evidence for a difference between an electronic option and postal only – perhaps due to electronic methods being more acceptable with increased computer access. The results in both subgroups were more homogeneous (I-squared = 48.5% and 7.0% respectively). The statistical test of homogeneity of the pooled effects in these two subgroups (*p* = 0.04) provides some evidence for different effects by year of study (Supplementary Fig. 3a). If we consider the most recent trials to better represent the situation today (i.e., greater access to computers than prior to 2012), then our best estimate of the effect on response of providing an electronic response option is from a synthesis of the studies published after 2012 (OR 1.01; 95% CI 0.93 to 1.08), i.e., no evidence for an effect.

##### Risk of bias

There was one study at high risk of bias [34]. Its results were entirely consistent with the results of the seven studies at low risk of bias (the statistical test of homogeneity of the pooled effects in these two subgroups was not significant (*p* = 0.454), Supplementary Fig. 3b).

### Results of assessments of evidence for reporting bias

#### Comparison 1 - postal vs. electronic questionnaire

There was no evidence for small study effects (Harbord’s modified test *p* = 0.148).

#### Comparison 2 - electronic questionnaire only vs. choice (postal or electronic)

There was no evidence for small study effects (Harbord’s modified test *p* = 0.841).

#### Comparison 3 - choice (electronic or postal) vs. postal questionnaire only

There was no evidence for small study effects (Harbord’s modified test *p* = 0.139).

## Discussion

### General interpretation of the results in the context of other evidence

This study has shown that response to a postal questionnaire is more likely than response to an electronic questionnaire. It has also shown that response is more likely when providing the option for postal response with an electronic questionnaire. It has further shown that providing an electronic response option with a postal questionnaire has no effect on response. Response is thus increased using postal rather than electronic questionnaires.

A previous meta-analysis of 43 mixed-mode surveys from 1996 to 2006 also found paper and postal administration produced greater response than electronic administration [50]. Our result that providing an electronic response option to postal administration does not increase response is consistent with a previous meta-analysis of randomised trials that found that mailed surveys that incorporate a concurrent Web option have significantly lower response rates than those that do not [51]. 

We suggest two possible reasons for these results:

- *Paper questionnaires are more accessible than electronic questionnaires*.

Although access to the Internet increased over the period during which the studies included in this study were conducted [5, 52], a ‘digital divide’ [53] persists in many populations where completion of a paper questionnaire may be possible, but completion of an electronic one may not.

- *Paper questionnaires are more personal than electronic questionnaires*.

Personalised materials have been shown to increase response [54]. If participants perceive a paper questionnaire with a return envelope to be more ‘personal’ than a request to go to a website to answer some questions, we should expect a higher response with paper.

### Strengths and limitations

The main strengths of this study are that our results are based on syntheses of the results of 45 randomised controlled trials that span two decades, and most of which were judged to be at low risk of bias.

There was, however, considerable heterogeneity between the results of the included studies. Our subgroup analyses did not identify any causes of heterogeneity among study results, but they did reveal confounding of the pooled result for postal versus electronic questionnaires. The unexplained heterogeneity means that we cannot be confident about the *magnitude* of the effects on response using postal rather than electronic questionnaires. However, from inspection of the forest plots we can be confident about the *direction* of these effects.

The evidence included in this review addresses ‘unit’ non-response only (i.e., return of questionnaires). ‘Item’ response (i.e., completion of individual questions) may be greater with electronic methods, but this was not addressed in this review and requires investigation in the future.

We assessed evidence for reporting bias using Harbord’s modified test for small-study effects and found no evidence for bias. This test may not be reliable given the substantial heterogeneity between the results of the included trials [55]. 

Due to the nature of this study (secondary analysis of a published review), there is no pre-registered protocol for the subgroup analyses provided in this study.

### Implications for practice, policy, and future research

These results will help researchers and healthcare providers to improve data collection from study participants and patients, helping to maintain study power and reduce bias due to missing data in research studies. In addition to the methods already known to be effective in increasing questionnaire response [8, 56], postal questionnaires should be used in preference to, or offered in addition to, electronic modes as this will help to increase the proportion of participants that responds. It should be noted, however, that the evidence upon which this recommendation is based is from studies published between 2001 and 2020, and this may change in the future as access to the Internet increases and more people become ‘tech-savvy’. Furthermore, we consider that the certainty of the evidence provided in this study is “Moderate”, due to the unexplained heterogeneity between the results of the included studies.

### Future research

Evidence on effective data collection in low- and middle-income settings is needed. Research centres such as LSHTM can embed studies within trials (SWATs) in their research in these settings to help to increase the evidence base [57]. 

Participation rates for epidemiologic studies have been declining [58]. Our study has presented evidence that postal questionnaires are preferable to electronic questionnaires to improve participation, but it does not tell us *why*. Research is still needed to advance sociological and psychological theories of participation in data collection procedures [59]. 

Electronic administration provides benefits for researchers over paper administration which have not been addressed by this study: A well-designed Web questionnaire can control skip patterns, check for allowable values and ranges and response consistencies, and it can include instructions and explanations about why a question is being asked [60]. These options could help to improve the completeness and quality of self-administered data collection, maintaining study power, reducing the risk of bias in study results, and saving study resources. Further research into the cost-effectiveness of electronic administration compared with postal administration in different settings will be needed to inform practice [61]. 

## Electronic supplementary material

Below is the link to the electronic supplementary material.

Supplementary Material 1

Supplementary Material 2

Supplementary Material 3

Supplementary Material 4

Supplementary Material 5

Supplementary Material 6

Supplementary Material 7

## Data Availability

Data extracted from included studies will be available in the forthcoming update on the Cochrane Library.
