# Supplementary figures and images for: Response is increased using postal rather than electronic questionnaires – new results from an updated Cochrane Systematic Review
Source: BMC Med Res Methodol. 2024 Sep 16;24:209. doi: 10.1186/s12874-024-02332-0 (PMC11403848; doi:10.1186/s12874-024-02332-0)

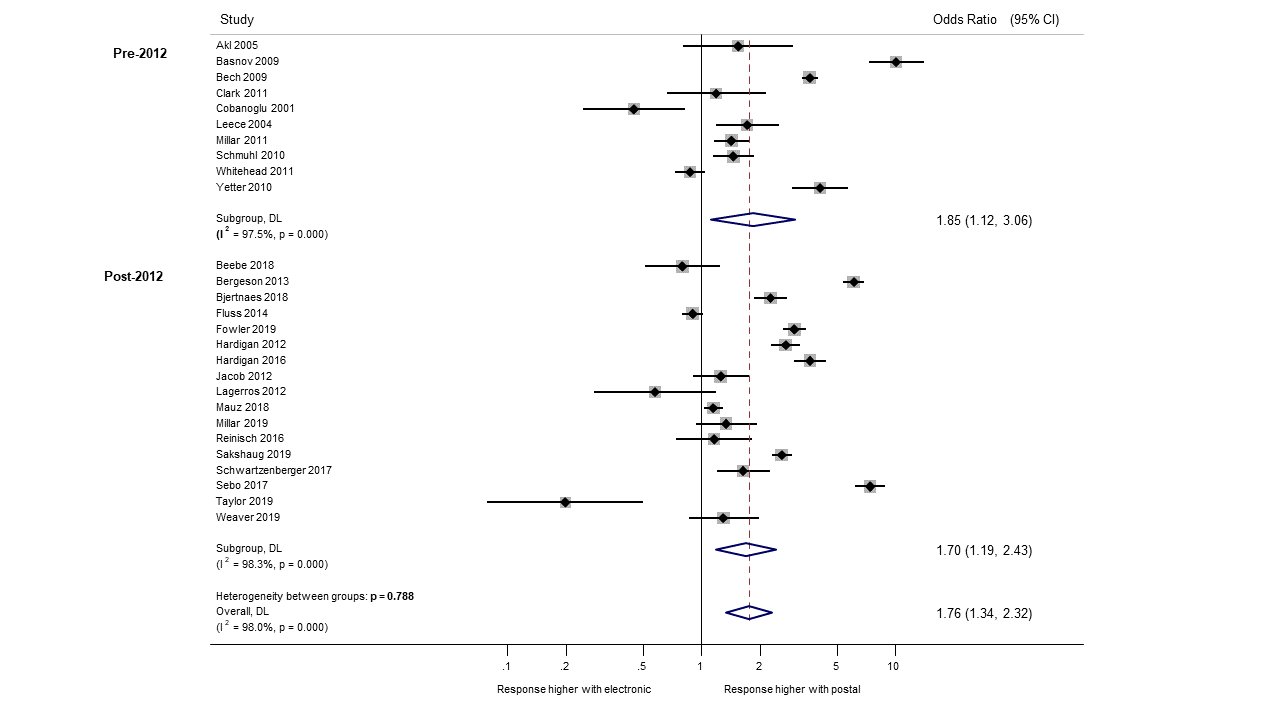

Supplement: Supplementary file 1 — Supplementary Material 1 [file 12874_2024_2332_MOESM1_ESM.png]

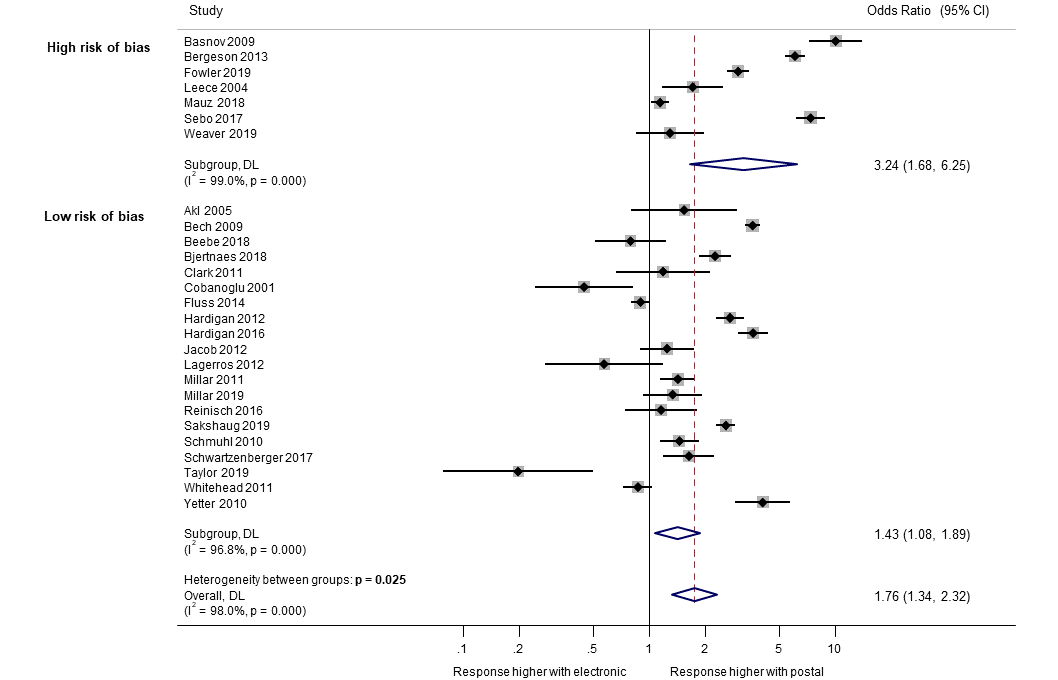

Supplement: Supplementary file 2 — Supplementary Material 2 [file 12874_2024_2332_MOESM2_ESM.png]

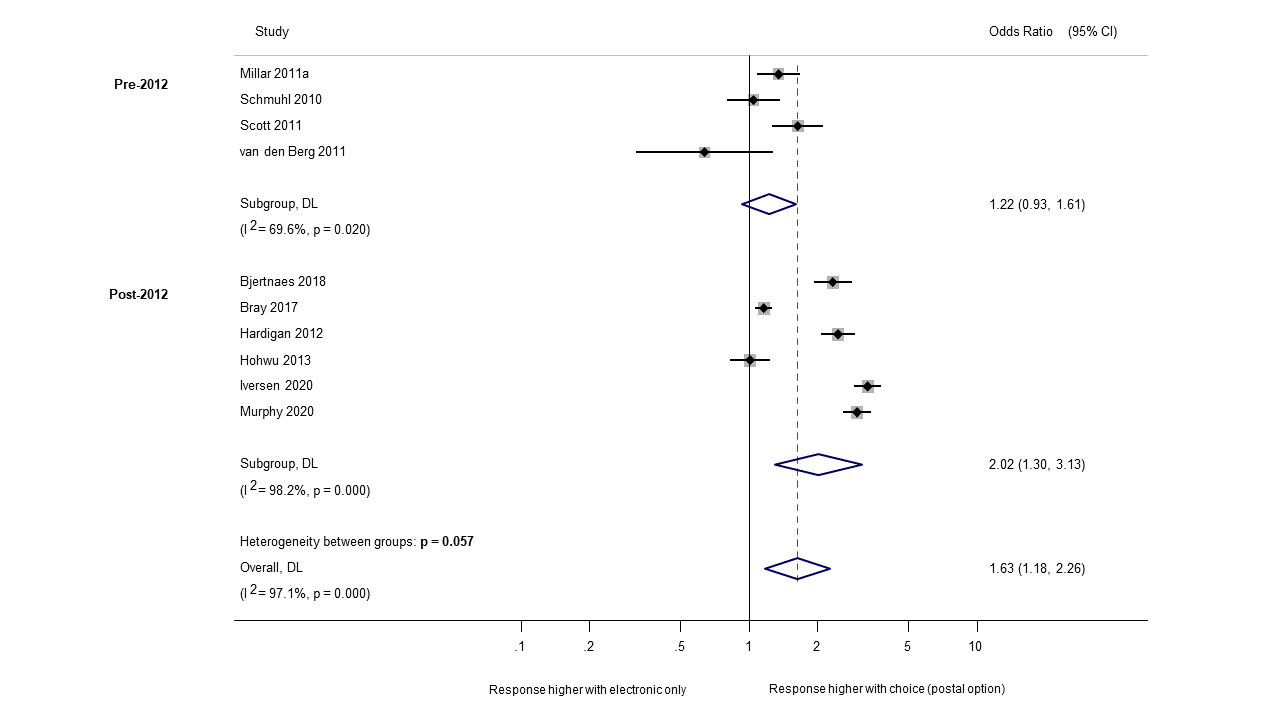

Supplement: Supplementary file 3 — Supplementary Material 3 [file 12874_2024_2332_MOESM3_ESM.png]

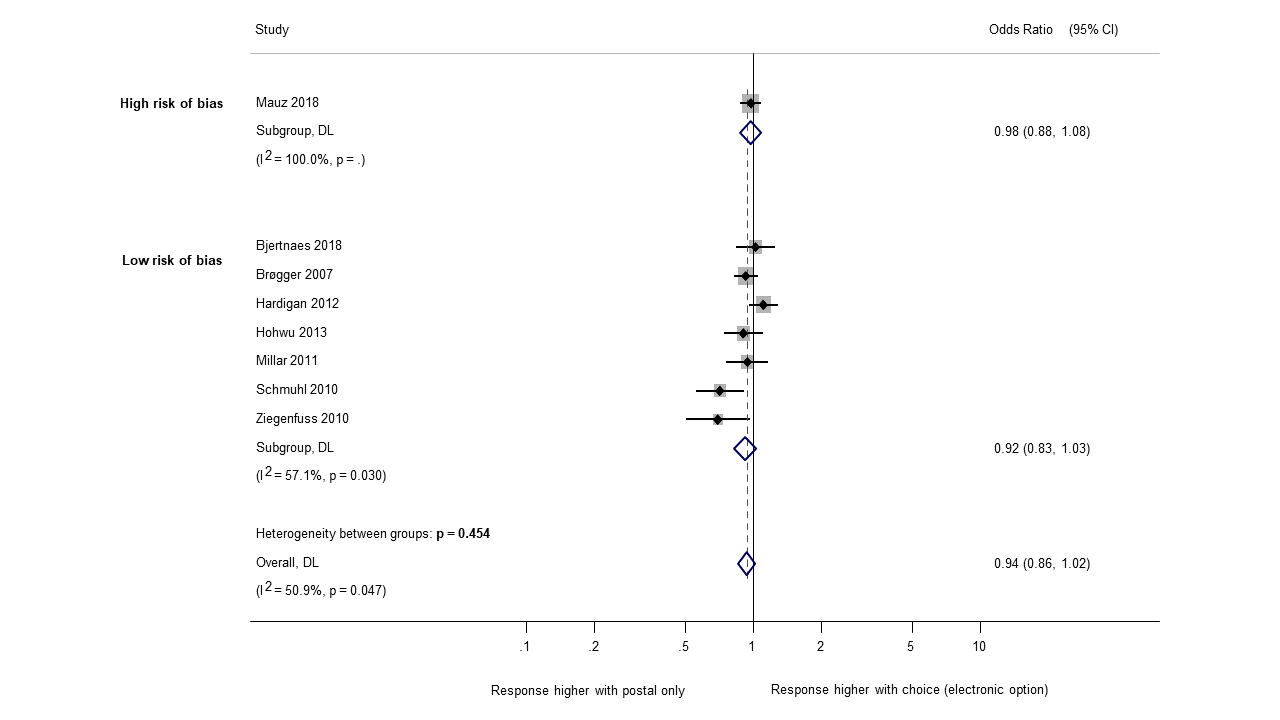

Supplement: Supplementary file 4 — Supplementary Material 4 [file 12874_2024_2332_MOESM4_ESM.png]

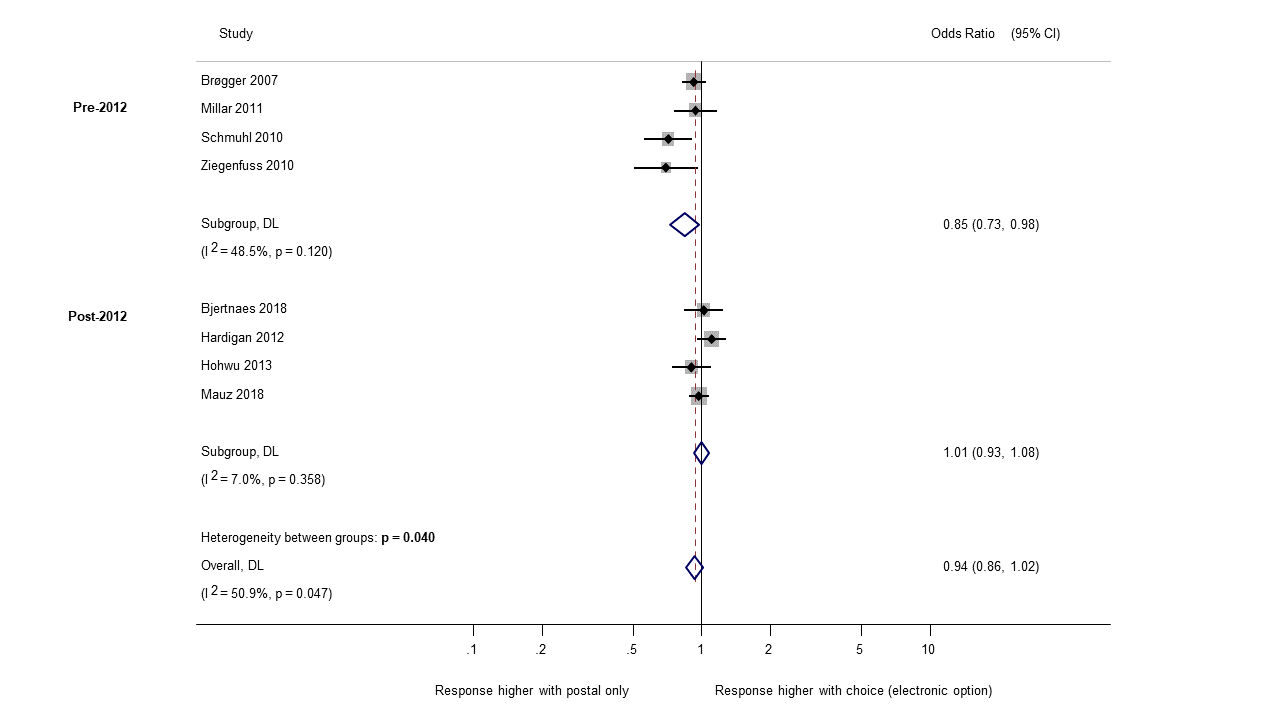

Supplement: Supplementary file 5 — Supplementary Material 5 [file 12874_2024_2332_MOESM5_ESM.png]

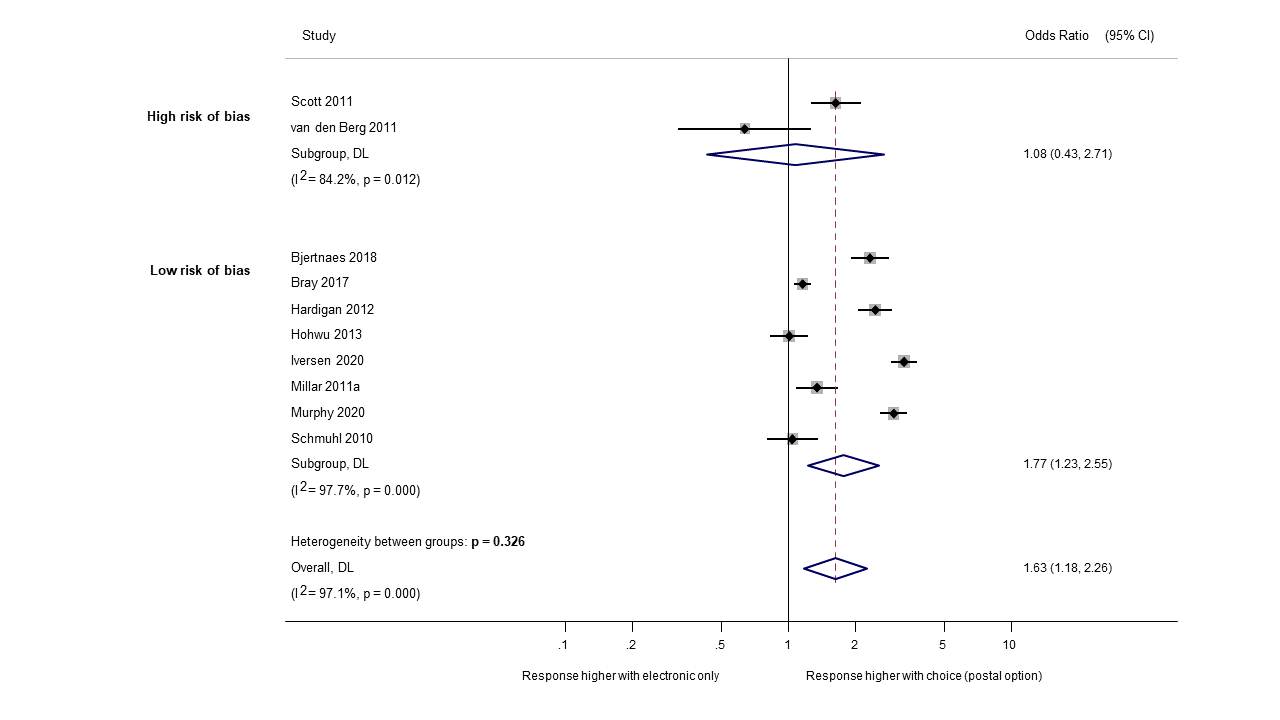

Supplement: Supplementary file 6 — Supplementary Material 6 [file 12874_2024_2332_MOESM6_ESM.png]
