# Supplementary material for: Response is increased using postal rather than electronic questionnaires – new results from an updated Cochrane Systematic Review
Source: BMC Med Res Methodol. 2024 Sep 16;24:209. doi: 10.1186/s12874-024-02332-0 (PMC11403848; doi:10.1186/s12874-024-02332-0)
Supplement: Supplementary file 7 — Supplementary Material 7 [file 12874_2024_2332_MOESM7_ESM.docx]

Supplementary figure titles

Supplementary Figure 1a. Effect on response of mode of administration in subgroups of year of study publication

Supplementary Figure 1b. Effect on response of mode of administration in subgroups of risk of bias (high, low)

Supplementary Figure 2a. Effect on response of choice of response mode compared with electronic only in subgroups of year of study publication

Supplementary Figure 2b. Effect on response of choice of response mode compared with electronic only in subgroups of risk of bias (high, low)

Supplementary Figure 3a. Effect on response of choice of response mode compared with postal only in subgroups of year of study publication

Supplementary Figure 3b. Effect on response of choice of response mode compared with postal only in subgroups of risk of bias (high, low)
